# Ubiquitin specific peptidase 3: an emerging deubiquitinase that regulates physiology and diseases

**DOI:** 10.1038/s41420-024-02010-6

**Published:** 2024-05-21

**Authors:** Yizhu Wang, Yanlong Shi, Kaiyi Niu, Rui Yang, Qingpeng Lv, Wenning Zhang, Kun Feng, Yewei Zhang

**Affiliations:** Hepatopancreatobiliary Center, The Second Affiliated Hospital of Nanjing Medical University, Nanjing, Jiangsu 210003 China

**Keywords:** Tumour biomarkers, Ubiquitylation

## Abstract

Proteins are the keystone for the execution of various life activities, and the maintenance of protein normalization is crucial for organisms. Ubiquitination, as a post-transcriptional modification, is widely present in organisms, and it relies on the sophisticated ubiquitin-proteasome (UPS) system that controls protein quality and modulates protein lifespan. Deubiquitinases (DUBs) counteract ubiquitination and are essential for the maintenance of homeostasis. Ubiquitin specific peptidase 3 (USP3) is a member of the DUBs that has received increasing attention in recent years. USP3 is a novel chromatin modifier that tightly regulates the DNA damage response (DDR) and maintains genome integrity. Meanwhile, USP3 acts as a key regulator of inflammatory vesicles and sustains the normal operation of the innate immune system. In addition, USP3 is aberrantly expressed in a wide range of cancers, such as gastric cancer, glioblastoma and neuroblastoma, implicating that USP3 could be an effective target for targeted therapies. In this review, we retrace all the current researches of USP3, describe the structure of USP3, elucidate its functions in DNA damage, immune and inflammatory responses and the cell cycle, and summarize the important role of USP3 in multiple cancers and diseases.

## Facts

- USP3 regulates the DNA damage response, and USP3 deficiency results in impaired cytogenetic stability.
- USP3 is involved in cell cycle regulation and affects cell division and proliferation.
- USP3 plays different roles in different cancers and functions as an oncogene in most cancers.

## Open Questions

- There are no targeted therapeutic agents or inhibitors for USP3, an area where current research is lacking.
- Research on USP3 in cancer is still limited to cellular and animal models, and further clinical trials are necessary.
- The regulatory mechanisms regarding USP3 are not sufficiently detailed and complete, and further in-depth investigation may be required in the future.

## Introduction

Ubiquitination, a widespread post-translational modification, plays a crucial role in regulating cellular functions, with the majority of cellular proteins undergoing ubiquitination [1]. The ubiquitin molecule consists of 76 amino acids that exhibit a high degree of conservation throughout evolutionary processes [2]. Ubiquitin attaches covalently to lysine residues on the substrate through a series of enzymatic reactions involving ubiquitin-activating (E1s), ubiquitin-conjugating (E2s), and ubiquitin ligase (E3s) enzymes [3–5], resulting in mono-ubiquitination (single ubiquitin) and poly-ubiquitination (ubiquitin chains) [6]. The ubiquitin-proteasome system (UPS) governs the degradation of over 80% of intracellular proteins, oversees protein quality control, and upholds protein homeostasis [7].

**Fig. 1:**
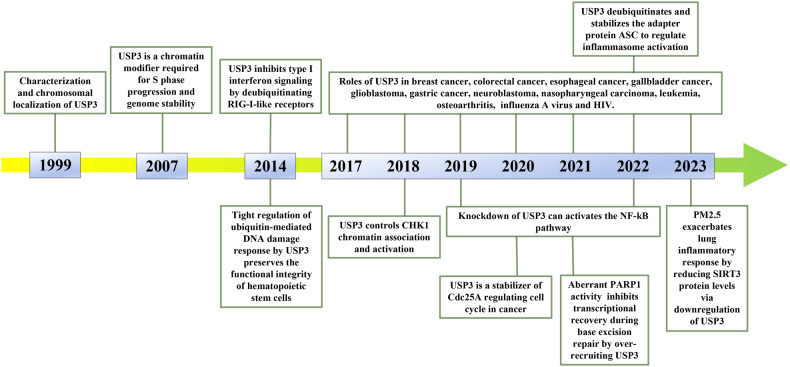
Timeline of USP3 research. A number of studies of significance and relevance to disease and signalling pathways are listed to show the broad framework of USP3 research.

Ubiquitination is a reversible process that is accomplished by deubiquitinases (DUBs). The approximately 100 DUBs in the human body are categorized into nine families [8]: ubiquitin‐specific proteases (USPs), ovarian tumor proteases (OTUs), ubiquitin C‐terminal hydrolases (UCHs), Machado‐Joseph domain‐containing proteases (MJDs, also known as Josephins), JAMM/MPN domain‐associated Zn‐depend metalloproteases (JAMMs, also known as MPN+), the motif interacting with ubiquitin (MIU)‐containing novel DUB family (MINDYs), monocyte chemotactic protein‐induced proteins (MCPIPs), permuted papain fold peptidases of dsRNA viruses and eukaryotes (PPPDEs), and zinc finger (ZnF) containing ubiquitin peptidase 1 (ZUP1) [9]. A burgeoning volume of researches has demonstrated that dysregulation of the balance between ubiquitination and deubiquitination can lead to a wide variety of adverse consequences [10]. USP3 is a member of the USPs, the largest family in DUBs. As the study of USP3 continues, researchers have found that USP3 is involved in a variety of physiological and pathological processes in cell. In the realm of innate immunity, USP3 plays a role in dampening antiviral responses by impeding the activation of the type I interferon (IFN) pathway through the elimination of Lysine 63 (K63)-linked polyubiquitin chains on RIG-I [11]. Within the context of renal cell carcinoma (RCC), the tumor suppressor E74-like transcription factor 5 has the ability to hinder the progression of RCC by promoting USP3-mediated deubiquitination and stabilization of WD40 and tetratricopeptide repeats 1 [12]. The multifaceted role of USP3 in various physiological processes underscores the importance of comprehensively investigating its structure, functions, and regulatory mechanisms, as such insights may offer novel therapeutic opportunities in the future. This review provides a comprehensive overview of the structure, functions, regulation and current research on USP3 in tumors and other diseases, and discusses the gaps and prospects for USP3 research. Particularly, the timeline of USP3 is listed in Fig. 1.

## The structure of USP3

**Fig. 2:**
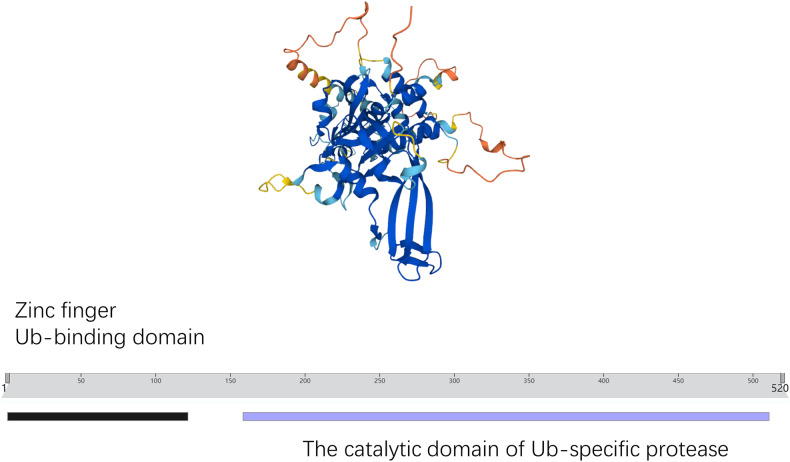
The structure and domains of USP3. Amino acids 1-121 form the Ub-binding domain Zinc finger and amino acids 159-511 comprise the catalytic domain of Ub-specific protease.

USP3, located on human chromosome 15q22.31, contains 520 amino acid residues. USP3 contains two conserved protein domains: the ZnF domain and the catalytic domain (UCH) [13]. Amino acids 1–121 constitute the UBP ZnF, whereas amino acids 159–511 constitute the UCH. The structure of the UBP ZnF domain is unique and does not resemble any other known structure. It has a compact globular fold with a deep cleft and a pocket accommodating the C terminus of ubiquitin (Fig. 2) [14]. The core catalytic structure of USPs is generally composed of three subdomains, which have been likened to the palm, thumb, and finger. The “fingers” are responsible for grabbing ubiquitin and the catalytic center is between the “palm” and the “thumb” [15]. The catalytic core domain of USPs contains a conserved cysteine catalytic triad, and the extended finger domain, together with the palm and thumb domains, forms the binding pocket of ubiquitin that recognizes the extended tail of ubiquitin and presents its c terminus to the active site cysteine [16]. ZnF and UCH jointly function, both are indispensable. Experiments confirmed that mutation of UBP ZnF significantly reduced the interaction of USP3 with ubiquitinated histone uH2A both in vivo and in vitro, suggesting that this is the major domain mediating the interaction between USP3 and ubiquitin [17]. In addition, USP3 was found to inhibit type I IFN signaling by deubiquitinating RIG-I-like receptors. However, the ZnF domain of USP3 alone cannot inhibit RIG-I-induced type I IFN activation, and the UCH domain may require ZnF for maximal catalytic activity, suggesting that both intact ZnF and the catalytic domain are required for USP3 to exert its deubiquitination function [11]. USP3 has the capability to interact with Claspin via the UCH domain for the purpose of deubiquitinating it, resulting in the activation of Claspin-dependent ATR-Chk1 signaling that ultimately contributes to the augmentation of glioblastoma radiation resistance [18]. In summary, the biological activity of USP3 is contingent upon the structural integrity of its UBP ZnF and UCH domains, which work in tandem to fulfill the physiological role of USP3.

## The functions of USP3

USP3 is a cysteine protease involved in a variety of physiological activities and perturbations in its function can result in adverse consequences. USP3 is implicated in DNA repair, cell cycle regulation, and apoptosis, with a particular focus on describing its role in maintaining genome stability and modulating inflammation.

## In genome stability

Genomic instability is deemed to be a hallmark of cancer [19]. DNA is vulnerable to alterations caused by external and internal factors, leading organisms to safeguard the genome via the DNA damage response (DDR) and cell-cycle checkpoints [20]. The DDR comprises two main signaling pathways: ATR-CHK1 and ATM-CHK2, with checkpoint kinase 1 (CHK1) being activated by phosphorylation from the upstream kinase ATR [21]. The interaction between USP3 and CHK1 results in the direct removal of the inhibitory ubiquitin chain at K63, leading to the release of CHK1 from chromatin and subsequent down-regulation of USP3, which enhances gene stability [22]. Histone ubiquitination is essential for the activation of DDR, and USP3 acts as a chromatin modifier by deubiquitinating monoubiquitinated H2A (uH2A) and H2B (uH2B) in vivo. The ZnF domain and uH2A are identified as the major structural domains and substrates, respectively, for USP3-mediated deubiquitination [17]. Further studies revealed that in addition to uH2A, USP3 also deubiquitinates conjugate Ub-γH2AX (variant H2A) at K13-15. In addition, USP3 regulates the aggregation of DDR factors (BRCA1 and 53BP1) at DNA damage sites, and overexpression of USP3 impairs the recruitment of these factors [23]. USP3 regulates mitotic progression, and USP3 deficiency induces DNA damage leading to replication defects and S-phase delays as well as activation of the corresponding checkpoints [17]. USP3 deubiquitinates and stabilizes the cell division cycle 25A (Cdc25A), a bispecific phosphatase that regulates the cell cycle, and degradation of Cdc25A in the presence of DNA damage causes cells to arrest in G1 phase [24]. Stem cell homeostasis is also critical for maintaining genomic stability. USP3-deficient mouse models exhibit severe haematopoietic impairment and hematopoietic stem cell (HSC) defects, with a decline in lymphocytes as they age, particularly in B-cell lines and T-cell lines. USP3 shields HSC from ionizing radiation (IR) and defends against spontaneous DNA damage in vivo via ubiquitin-dependent DDR. Deficiency of USP3 makes HSC more sensitive to genotoxicity and contributes to severe genomic rearrangements due to spontaneously generated DSB-triggered repair pathways [25].

## In inflammatory vesicles

Inflammatory vesicles are integral to the innate immune system, aiding in immune homeostasis, with NLRP3 inflammasomes being the subject of extensive research [26]. Apoptosis-associated speck-like protein containing a caspase recruitment domain (ASC) functions as a central bridging protein for multiple inflammatory vesicles, with USP3 playing a role in enhancing ASC stability through the removal of K48-linked ubiquitin chains. The formation of ASC speckles is indicative of NLRP3 inflammatory vesicle activation, with USP3 knockdown resulting in a significant reduction in the number of ASC speckles. Furthermore, in addition to NLRP3, USP3 overexpression in vivo also facilitates the activation of AIM2 and NLRC4 [27]. USP3, serving as a regulator of inflammatory vesicle activation, plays a crucial role in the precise control of inflammation and the maintenance of immune homeostasis.

## Signaling pathways in USP3

USP3 is engaged in regulating multiple signaling pathways, such as type I IFN, NF-κB, and PI3K/AKT signaling pathways. This review specifically focuses on elucidating the mechanisms involved in the type I IFN and NF-κB pathways, which are crucial components of the innate immune system and are activated upon detection of pathogenic molecules by pattern recognition receptors (PRRs), such as Toll-like receptors (TLRs), Nod-like receptors (NLRs), and RIG-I-like receptors (RLRs) [28].

## Type I IFN signaling

Type I IFN, a cytokine produced in response to viral infection, is generated through PRR-mediated mechanisms. RLRs serve as intracellular PRRs for pathogen detection, with humans possessing three RLRs: RIG-I, melanoma differentiation-associated gene 5 (MDA5), and LGP2 [29, 30]. The N-terminal caspase activation recruitment domain (CARD) of RIG-I and MDA5 interacts with the mitochondrial protein MASV (mitochondrial antiviral signaling) in a ligand-dependent manner. USP3 modulates the type I IFN response by specifically binding to the CARD domains of RIG-I and MDA5, thereby inhibiting their activation. USP3 selectively cleaves the K63-linked ubiquitin chains on RIG-I, which are crucial for initiating the type I IFN response, without affecting ubiquitin chains on other signaling proteins [11]. Influenza A virus (IAV), which has caused several world pandemics, is highly pathogenic to humans, and the IFN pathway is one of the effective ways to combat it. USP3 is a negative regulator of the type I IFN pathway, and miR-26a can attack USP3 to activate the IFN pathway and thus inhibit IAV infection [31].

## NF-κB signaling

In addition to its role in the type I IFN pathway, USP3 also negatively regulates the NF-κB signaling pathway induced by TLRs. USP3 plays a critical role in inhibiting NF-κB activation induced by TLR and IL-1R signaling through cytoplasmic translocation and cleavage of K63-linked ubiquitin chains on the adapter protein MyD88. USP3 is a key component of the MyD88-USP3 axis, essential for the development of innate immune tolerance [32]. In osteoarthritis, digoxin can directly target the miR-146b-5p/Usp3&Sox5 pathways to inhibit macrophage M1-like polarization, which improves the arthritic microenvironment [33]. But surprisingly, USP3 plays a positive role in the fight against human immunodeficiency virus (HIV). USP3 can deliver HIV-1 inhibition in two ways, either in an enzyme-dependent or enzyme-independent manner [34]. Vif is a viral accessory protein encoded by HIV that induces degradation of APOBEC3G (A3G), a host defensive factor. In the presence of Vif, USP3 reduces A3G degradation by deubiquitinating Vif-induced polyubiquitination in an enzyme-dependent manner. At the same time, USP3 can directly stabilize A3G mRNA to increase the amount of A3G. The experiments showed a positive correlation between USP3 mRNA, A3G mRNA and the number of CD4+ T cells [34].

## USP3 in cancers

**Fig. 3:**
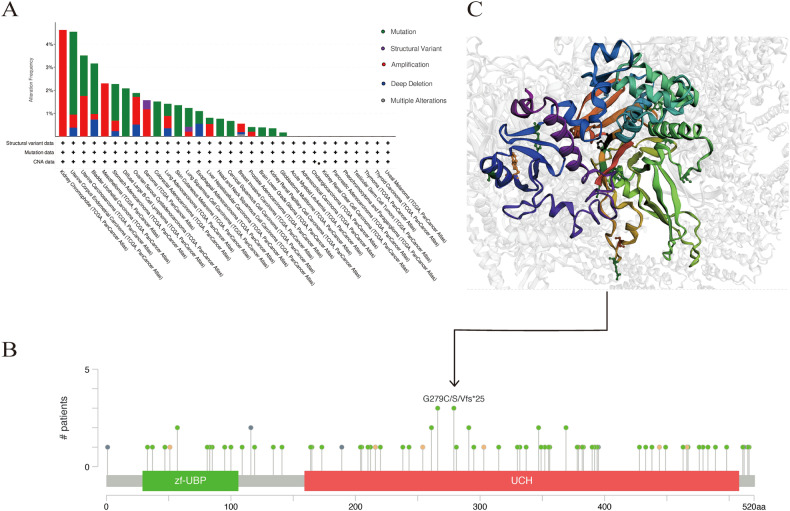
Mutation features of USP3. **A** Mutation type in different tumors of TCGA; **B** mutation site; **C** the 3D structure of the mutation site (G279C/S/Vfs).

**Fig. 4:**
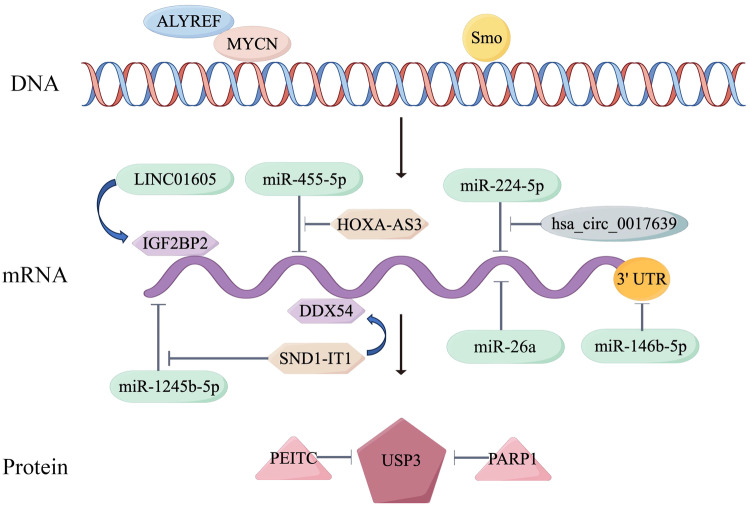
Regulation of USP3 expression in different levels. The formation of a transcriptional activation complex involving ALYREF and MYCN and Smo promote the transcription of USP3 in the DNA level; related ceRNA network of USP3 and RNA-binding proteins regulate USP3 expression in the mRNA level; PEITC (Phenethylisothiocyanate, a DUB inhibitor) and PARP1 (poly-ADP-ribose polymerase 1, a positive role) can influence USP3 (drawn by Figdraw) in the protein level.

USP3 demonstrates abnormal expression in various cancer types and contains multiple mutation sites (Fig. 3), in addition to its involvement in the pathogenesis of several other diseases (Table 1). The regulatory mechanisms governing USP3 are outlined in Fig. 4.

**Table 1:** The roles of USP3 in a variety of diseases and interacting molecules.

Diseases	The role of USP3	Related molecule	References
Breast cancer	+	KLF5	Wu et al. [64]
Colorectal cancer	−	SMAD4	Wang et al. [67]
		AR	Zheng et al. [69]
Esophageal cancer	+	Aurora A	Shi et al. [62]
Gallbladder cancer	+	PKLR	Liang et al. [57]
Glioblastoma	+	Snail	Fan et al. [45]
		miR455-5p	Chen et al. [46]
		Smo	Tu et al. [18]
Gastric cancer	+	SND1-IT1	Jin et al. [40]
		COL9A3 COL6A5	Wu et al. (2021)
		miR-224-5p	Li et al. [39]
		SUZ12	Wu et al. [64]
		/	Fang at al. [37]
Human immunodeficiency virus-1	−	A3G	Zhao et al. [34]
Influenza A virus	+	miR-26a	Gao et al. [31]
Leukemia	−	H2AK119ub	Chae et al. [65]
Neuroblastoma	+	ALYREF	Nagy et al. [50]
		REST	Karapurkar et al. [52]
Nasopharyngeal carcinoma	+	LINC01605	Zhao et al. [55]
Non-small cell lung cancers	+	RBM4	Liao et al. [59]
Osteoarthritis	−	miR-146b-5p	Jia et al. [33]

+ promotion role, − inhibition role.

## In gastric cancer (GC)

GC is ranked as the fifth most prevalent malignant disease globally, resulting in the third highest number of cancer-related fatalities [35]. Increasing evidence suggests a significant role for USP3 in the progression of gastric cancer. The upregulation of USP3 in GC has been found to significantly enhance cell proliferation in vitro and promote metastasis in vivo [36, 37]. Increased expression of USP3 is associated with decreased 5-year overall survival rates in late-stage GC (stages III and IV), while no impact is observed in early-stage GC (stages I and II) [37]. Additionally, USP3 has been shown to facilitate migration and invasion by contributing to epithelial-to-mesenchymal transition (EMT) induced by TGF-β1 in GC [36]. Furthermore, researches show that SUZ12 [36], COL9A3 and COL6A5 [38] are differentially expressed proteins that may serve as potential interaction targets of USP3, contributing to the migration and invasion processes in GC by participating in the EMT. USP3 plays a role in stabilizing the protein levels of these proteins through deubiquitination and interaction, with protein level regulation occurring exclusively at the post-transcriptional stage. Notably, the expression of USP3 is positively correlated with SUZ12, COL9A3 and COL6A5 protein expression levels. Meanwhile, USP3 is capable of influencing the proliferation and migration of GC cells by modulating the hsa_circ_0017639/miR-224-5p/USP3 axis as described above [39]. The latest study demonstrates that GC cells can induce malignant transformation of GES-1 cells through USP3 by secreting SND1-IT1, an exosome that is oncogenic in vivo, into GES-1 cells. The regulatory mechanism not only relies on the SND1-IT1/miR-1245b-5p/USP3 axis, but SND1-IT1 also recruits DDX54 to stabilize USP3, while USP3 also mediates the deubiquitination of SNAIL1 [40]. Taken together, USP3 overexpression is an independent prognostic marker for GC [41].

## In glioblastoma (GBM)

GBM is identified as the most aggressive form of gliomas and one of the prevalent brain tumors, exhibiting a median survival rate of merely 14–16 months [42, 43]. Furthermore, it is recognized as the most common malignant tumor affecting the central nervous system [44]. USP3 displays significant upregulation in both tissues and cell lines within GBM, correlating with a decreased overall survival rate. In GBM, the transcription factor Snail plays a crucial role in USP3-mediated EMT, promoting cell invasion, migration, and tumor progression [45]. A study conducted by Tu et al. highlights that heightened expression of Smo often indicates a poor prognosis in GBM post-radiation therapy, with Smo contributing to radioresistance. Notably, this study finds that this effect is dependent on Claspin polyubiquitination and proteasomal degradation, which can be reduced by USP3 transcription, resulting in regulation of ATR-Chk1 signaling [18]. Another study by Chen et al. proposes that HOXA-AS3/miR455-5p/USP3 axis is involved in the regulation of GBM that the lncRNA HOXA-AS3 expression is elevated in GBM and significantly associated with a lower overall survival rate. The lncRNA HOXA-AS3 silence inhibits tumorigenicity and EMT process in GBM cells that provides a new direction for our understanding of GBM [46].

## In neuroblastoma (NB)

NB, a prevalent extracranial solid tumor in children originating from the sympathetic nervous system, is responsible for approximately 10% of pediatric cancer deaths [47, 48]. Research indicates that the MYCN-ALYREF-USP3 signaling pathway plays a crucial role in driving NB tumorigenesis. MYCN, a stable oncogenic transcription factor, is tightly regulated by the ubiquitin-proteasome system and partially relies on ALYREF for promoting enhanced cancer cell viability and proliferation [49], while ALYREF inhibits MYCN degradation by directly transcribing USP3 at levels necessary to facilitate tumor growth. MYCN is a direct target of USP3, and MYCN expression is positively correlated with USP3; high USP3 expression alone is not meaningful [50]. A recent article found that USP3 can also regulate tumor self-renewal and proliferative capacity by interacting with the repressor element-1 (RE-1) silencing transcription factor (REST) in neuroblastoma, further demonstrating that USP3 is a potential prognostic marker for neuroblastoma. USP3, one of the DUBs with the highest expression of USP family genes in neuroblastoma, is positively correlated with REST, a transcriptional repressor in non-neuronal cells and neural stem cells [51], and USP3 promotes tumor formation and growth by stabilizing the level of REST proteins through deubiquitination [52].

## In other cancers

USP3 exhibits aberrant expression in various cancers, suggesting its significant role in cancer progression. Nasopharyngeal carcinoma (NPC) has a relatively low incidence but is geographically specific and strongly linked to Epstein–Barr virus (EBV) infection [53, 54]. In NPC, the LINC01605/miR-942-5p/Ikbkb ceRNA network modulates cancer cell behavior, with LINC01605 facilitating the recruitment of IGF2BP2 to stabilize USP3. Furthermore, USP3 enhances Ikbkb protein levels by deubiquitinating Ikbkb. LINC01605 and NF-κB pathway activated by nuclear translocation of p65 promoted by the expression of Ikbkb form a positive feedback regulation [55]. Gallbladder cancer (GBC) is a rare yet highly aggressive malignancy, with surgery being the most efficacious treatment option, but only a small number of people are eligible for surgery [56]. Pyruvate kinase L/R (PKLR) is a key enzyme in glycolysis that drives tumor development [44]. USP3 deubiquitinates and stabilizes PKLR in GBC and is the positive regulator of PKLR to impact GBC cells [57]. Lung cancer, particularly non-small cell lung cancers (NSCLCs), is the leading cause of cancer-related mortality, representing approximately 85% of all lung cancer cases [58]. Within NSCLC, RNA Binding Motif 4 (RBM4) serves as a direct target of USP3, which modulates its activity to enhance the proliferation of NSCLC cells [59]. Esophageal cancer (ESCC) ranks as the eighth most prevalent cancer globally, with ESCC accounting for approximately 90% of cases. The prognosis for ESCC is notably unfavorable, as evidenced by 5-year survival rates ranging from 10 to 30% [60, 61]. Elevated levels of USP3 and Aurora A, both of which are deubiquitinated by USP3, have been observed in ESCC and are associated with increased cell proliferation, invasion, and metastasis [62]. Breast cancer is the most common malignant tumor in women and the second leading cause of cancer deaths in women [63]. The study of Wu et al. [38] describes that USP3 is the DUB of the Krüppel-like factor 5 (KLF5) in breast cancer and interacts with KLF5 in the nucleus. USP3 is dependent on KLF5 and ectopic expression of KLF5 partially rescues the proliferation inhibiting effect of USP3 knockdown in breast cancer cells in vitro and tumorigenesis in vivo. Notably, USP3 individually makes breast cancer cells resistant to drugs [64]. Interestingly, USP3 may play an integral role in acute myeloid leukemia as a tumor suppressor by regulating H2AK119u in 12-O-tetradecanoyl phorbol-13-acetate (TPA)-induced differentiation of HL-60 cells, which offers a prospective therapeutic approach [65]. Current studies in colorectal cancer (CRC) may be slightly contradictory. CRC has the third highest incidence rate among cancers, and most people are diagnosed at an advanced stage, with a five-year survival rate of less than 20% [66]. Contrary to the previously described cancers, USP3 is downregulated in CRC, and USP3 deficiency leads to tumor metastasis and poor prognosis which can be inhibited by overexpression of the USP3 3’UTR. USP3 forms a ceRNA network with miR-224, SMAD4 [67]. Oxaliplatin blocks DNA replication and transcription and is considered the standard drug for advanced CRC [68]. USP3 can also impact CRC sensitivity to oxaliplatin by mediating the USP3/AR/RASGRP3 axis. USP3 promotes transcription of RASGRP3 by deubiquitinating and stabilizing the transcription factor AR, and up-regulation of RASGRP3 increases CRC sensitivity to oxaliplatin [69]. However, research of Alkhizzi et al. reveals that both linear and circular isoforms of USP3 are upregulated in CRC. The linear USP3 is more prominently upregulated and there is a strong positive correlation between them in CRC patients, making linear USP3 a potential non-invasive CRC biomarker [70]. In recent years, an increasing number of studies have shown that USP3 is involved in the development of several tumors, and it is consequently essential to investigate the regulatory mechanisms of USP3 in cancer, which may provide us with evidence for the development of USP3 inhibitors. USP3 may serve as new prognostic markers and therapeutic targets in the future.

## Conclusion and perspective

Proteins are the basic units for performing life activities, and ubiquitination, as one of the most fundamental post-translational modifications, is relevant to all aspects of cell biology. USP3 undoubtedly has an important role as a member of the DUBs. After these two decades since USP3 was characterized in 1999, we have gained a preliminary understanding of the structure, physiological and pathological functions of USP3 and the role it plays in different diseases. However, research on USP3 is still relatively limited compared to other members of the USP family, and there are many areas where we have not yet set foot and need to continue to explore. We still have a lot of questions, for example, does USP3 have only one conformation? Does it have other isoforms, and can USP3 form complexes with other molecules to function?

Through the current research, we can find that USP3 plays similar or opposite roles in different types of cancers. For future research, perhaps we need to focus more on a specific cancer: activating or inhibiting USP3 to achieve better therapeutic effects. However, it is important to note that USP3 also plays a role in inflammatory activation and immune processes, and whether over-activation or inhibition of USP3 in order to achieve better efficacy may simultaneously lead to disturbances in the body’s immune regulation should be given additional attention in the therapeutic strategy. Similar to proteasome inhibitors, DUB inhibitors are moving into clinical trials, but no drugs have yet been approved for clinical application. There is still a blank field regarding USP3-specific inhibitors, and perhaps we should put more attention on developing DUB-specific inhibitors for better targeted therapy.
